# Variations in Soil Nitrogen Availability and Crop Yields under a Three-Year Annual Wheat and Maize Rotation in a Fluvo-Aquic Soil

**DOI:** 10.3390/plants12040808

**Published:** 2023-02-10

**Authors:** Runzhi Niu, Changwei Zhu, Guiying Jiang, Jin Yang, Xuanlin Zhu, Lianyi Li, Fengmin Shen, Xiaolei Jie, Shiliang Liu

**Affiliations:** College of Resources and Environmental Sciences, Henan Agricultural University, Zhengzhou 450002, China

**Keywords:** rotational tillage, soil nutrients, maize yield, fluvo-aquic soil, ecosystem multifunctionality

## Abstract

Optimum tillage practices can create a suitable soil environment, and they improve the soil nutrient status to ensure crop development and yield. In this study, we evaluated the influences of six tillage practices on soil nutrients and maize yields from 2017 to 2019 in fluvo-aquic soil in the North China Plain. The field experiment was carried out by a split design with rotary tillage (RT) and deep tillage (DT) in wheat season in the main plot and no-tillage (NT), subsoiling between the row (SBR), and subsoiling in the row (SIR) in maize season in the subplot. The results showed that the soil nutrient content was higher under the treatments with rotary tillage in the wheat season in the 0–20 cm soil layer, while in the 20–40 cm soil layer, the soil nutrient content was higher under the treatments with deep tillage in the wheat season. The integrated principal component scores indicated that the soil nutrients had improved in the second year. The ecosystem multifunctionality (EMF) index was higher with the treatments with rotary tillage in wheat season in the 0–20 cm soil layer, while it was the highest under DT-SIR at 20–40 cm. Correlation analysis showed that the soil EMF index correlated significantly (*p* < 0.05) with the soil nutrient content mainly in the 0–40 cm soil layer. The higher maize yield was under the treatments with deep tillage compared to that under the treatments with rotary tillage in the wheat season. The yield-increasing effect was higher under the treatments with subsoiling than those utilizing no-till in the maize season, with the highest average yield of 13,910 kg hm^−2^ in the DT-SIR during the three years. Maize yield was strongly correlated with nutrients in the subsoil layer. The higher yield stability was found under RT-NT. To sum up, during the three-year experiment, rotary tillage in the wheat season combined with subsoiling in the maize season improved the soil nutrient content and the EMF index in the 0–20 cm layer, while the combination of deep tillage in the wheat season and subsoiling in the maize season improved those indices in the 20–40 cm soil layer, and increased the maize yield, the best one was under DT-SIR.

## 1. Introduction

Tillage practice is the major agriculture management technique to change soil structure and regulate the soil ecological environment and soil nutrient cycling. The soil physical and chemical properties fluctuate based on the different tillage practices with their disturbing degrees. Meanwhile, different tillage modes directly affect soil microorganisms’ microenvironments and further regulate soil nutrient cycling. The optimum tillage practice can improve the soil water and heat conditions, further help boost the soil nutrient cycling, and increases the crop yield [1,2,3]. Soil microorganisms drive soil nutrient cycling. The changes in soil microorganism community structure and their extracellular enzyme activity are the critical indexes by which to assess soil quality, soil fertility, and soil nutrient cycling [4]. Meanwhile, these indexes will quickly respond to changes in tillage practices. Therefore, it is necessary to explore the effect of tillage practices on the soil nutrient cycle and soil microorganism functioning.

Studies have found that rotation tillage in the winter wheat season was an important practice that enriched the microbial community structure and increased the soil enzyme activity, thus facilitating the conversion and accumulation of soil nutrients and improving crop yields [2,5]. Deep tillage to 30 cm increased total soil N content in the 0–40 cm soil layer and improved crop root distribution [6], further promoting crop water efficiency and grain production [7]. Rotary and subsoiling tillage rotations combined with straw return could increase soil nutrient content and crop yield [8,9]. Liu et al. [10] reported that lower compaction and higher SOC were found under no-till combined with subsoiling (NS), which suggested that NS improved soil physicochemical properties. Subsoiling tillage was not only effective in the topsoil but also in the subsoil. This was confirmed by a study in China that found subsoiling to a depth of 35 cm increased the available soil nitrogen, phosphorus, and potassium contents in the 20–40 cm soil layer, contributing to an increase in annual yield [2,11]. Combined with the straw return, subsoiling tillage was effective in avoiding soil compaction and facilitating soil aggregation [12,13,14,15], while deep tillage could raise the soil fertility and increase the thickness of the cultivated horizon, further leading to higher SOC and TN [16,17,18]. However, these studies were focused on the wheat season and less on the maize season. For maize season, long-term no-till led to compaction in the subsoil, which seriously hindered the exchange of water, fertilizer, air, and heat and thus reduced soil quality and crop yield [19,20].

Compared with no-till in the summer maize season, subsoiling tillage effectively increased soil water content, dry shoot biomass, and the net photosynthetic rate, further increasing crop yield and decreasing production costs [19,20]. For the single-cropping system area, subsoiling in maize season not only increased the plow layer depth and benefited the maize root growth and physiological activity [21,22,23], but also improved the water retention capacity, crop water use efficiency, and crop yield [24]. The studies in wheat–maize crop rotation areas found that subsoiling tillage facilitated the long-term maintenance of soil quality to improve crop productivity, and wheat and maize straw return also played an instrumental role [25]. Subsoiling is a more common tillage practice. This practice creates a better soil structure where disturbed loose soil and undisturbed compact soil are juxtaposed longitudinally at certain depths and certain intervals [26].

The tillage practices directly change the soil physicochemical properties, further affect the soil nutrient cycle drivers and the soil microorganism community and structure, and, finally, change the ecosystem multifunctionality. Ecosystem multifunctionality (EMF) is described as the simultaneous provision of multiple functions and services by an ecosystem [27]. Soil multifunctionality (SMF) refers to the ability of soil ecosystems to provide and maintain multiple functions in parallel, including soil nutrient cycling, soil nutrient storage, and microbial activity [26], and is often used as one of the most important indicators to quantify soil ecosystem multifunctionality [28]. Soil diversity is strongly related to nutrient cycling [29]. Agricultural management, such as tillage and fertilizer application, affects the soil nutrient availability in arable land, which is an important factor in maintaining soil health [30,31].

The North China Plain (NCP) is the main agricultural production area in China, which contributes to about 25% of the national grain production [32]. The main cropping system in this area was wheat (*Triticum aestivum* L.)–maize (*Zea mays* L.) rotation [33]. Conventional tillage with rotary tillage in wheat season and no-tillage in maize season is the dominant tillage management practice. It results in a shallow plow layer with a compacted plow pan beneath the topsoil layer that restricts crop root growth, thereby affecting grain yields. Therefore, rotational tillage, which consists of a combination of no-till, rotary tillage, deep tillage, and subsoiling, has received substantial attention. Generally, previous studies about subsoiling concentrated on the single-cropping system or on the wheat season under a wheat–maize rotation system. Little research has focused on the effects of subsoiling tillage in maize season given a wheat–maize rotation system. Fluvo-aquic soil is the typical soil type in the NCP. The optimal rotational cropping combinations need further research on wheat–maize double-cropping systems in fluvo-aquic soil. Therefore, the objectives of this study were to (i) assess the effects on soil nutrient contents, ecosystem multifunctionality, and crop yield and its sustainability and (ii) identify suitable sustainable tillage management practices based on their effects on soil properties and crop productivity in fluvo-aquic soil in the North China Plain.

## 2. Results

### 2.1. Soil Nutrient Content

#### 2.1.1. Soil Organic Matter and Total Nitrogen Contents

**Figure 1:**
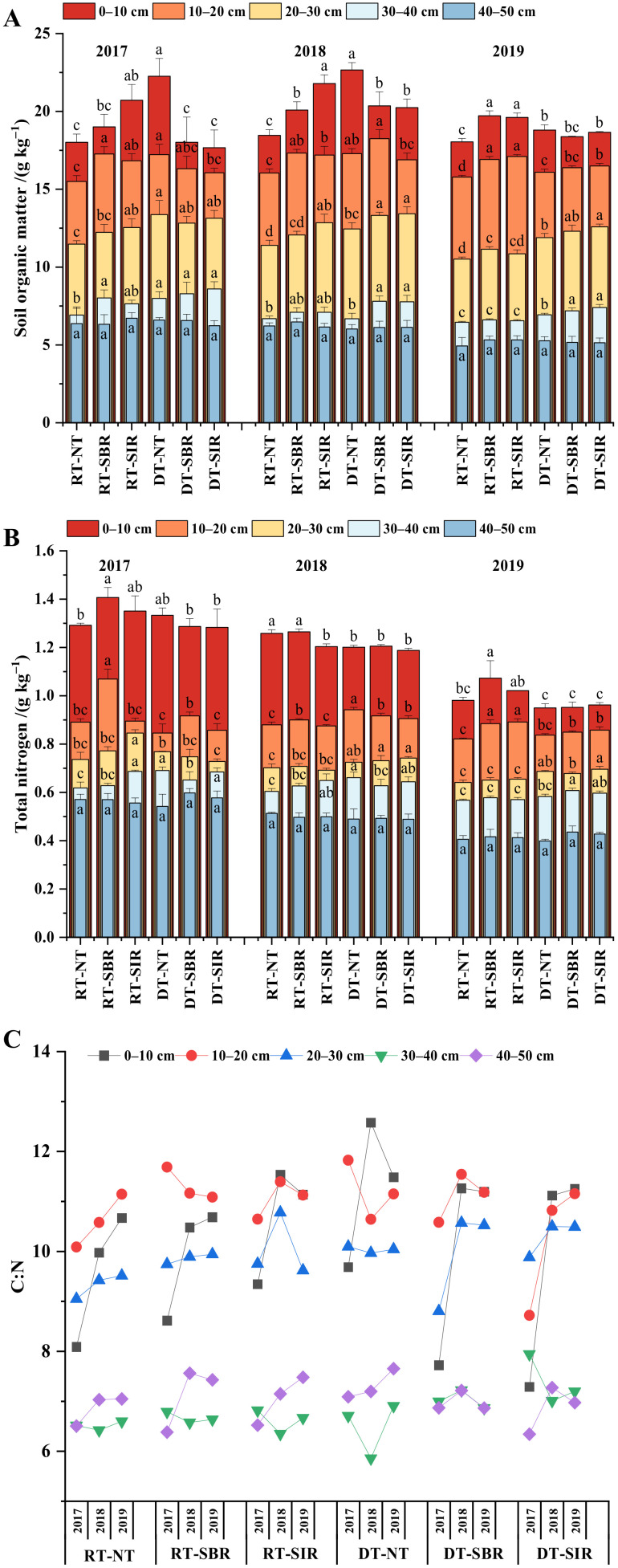
Soil organic matter (**A**), total nitrogen (**B**), C:N (**C**) under different treatments in different soil depths from 2017 to 2019 during the experiment. Bars represent standard error. Different lowercase letters above the bars represent significant differences among treatments (*p* < 0.05).

Soil organic matter (SOM, Figure 1A) and total nitrogen (TN) contents (Figure 1B) decreased with soil depth. They significantly differed among different tillage modes in 0–40 cm soil depths. The SOM and TN contents at 0–20 cm soil depth under RT-SBR and RT-SIR were higher than under the other treatments, while at 20–40 cm soil depth, the higher SOM and TN contents were found under the treatments with deep tillage in wheat season, with the highest values of 10.49 g kg^−1^ and 0.69 g kg^−1^ under DT-SIR and DT-NT, respectively. During the three-year experiment, the SOM content at 0–20 cm soil depth increased first and then decreased, with a maximum increment of 7.25% compared with 2017 under treatments with subsoiling in maize season. At 20–40 cm soil depth, the SOM content decreased with time. The TN content under all treatments decreased with time; the range of decrease was 0.04–40.62%.

The C:N ratio, SOM, and TN decreased with soil depth, and the first was higher than the others at 0–20 cm (Figure 1C). Compared with the treatments with rotary tillage, the C:N ratio at 20–40 cm soil depth increased under treatments with deep tillage in wheat season. Moreover, the C:N ratio under DT-SIR increased by an average of 7.11% during the three years compared to RT-SIR.

#### 2.1.2. Soil Alkaline Nitrogen, Available Phosphorus, and Available Potassium Contents

**Figure 2:**
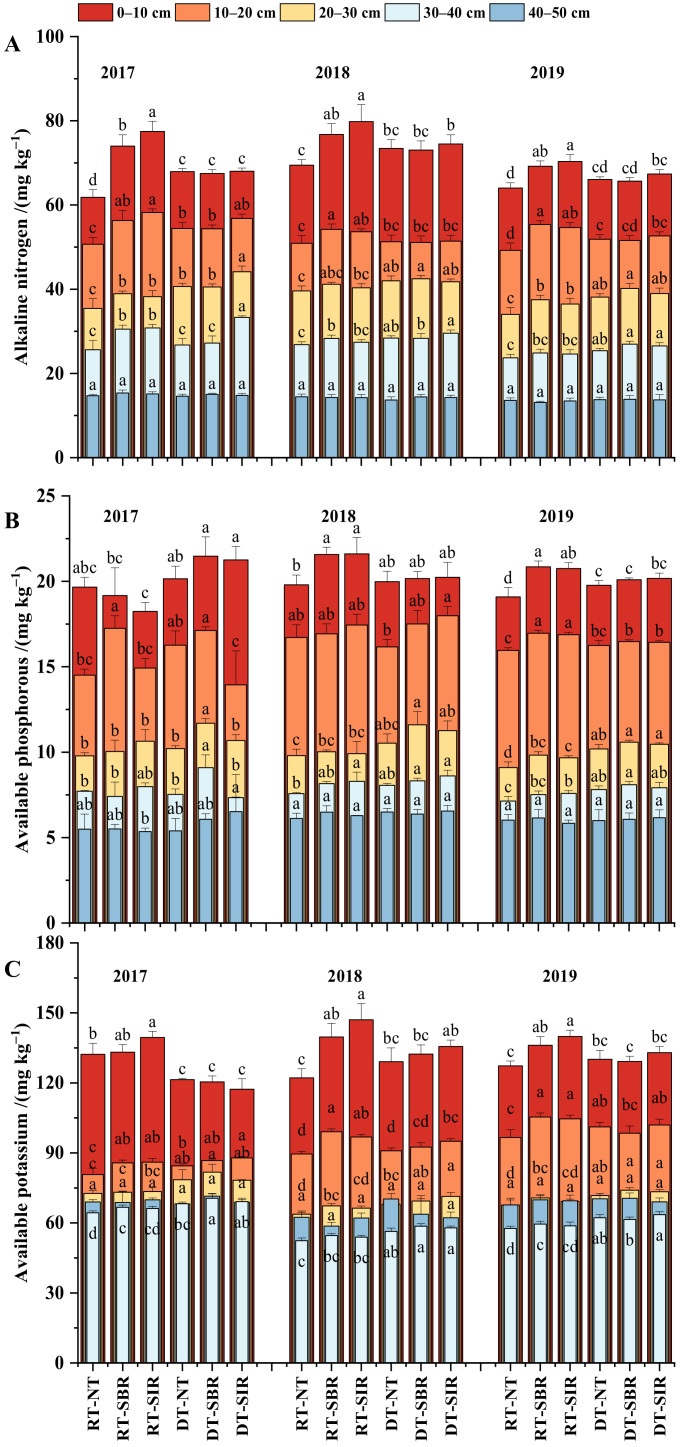
Soil alkaline nitrogen (**A**), available phosphorous (**B**), and available potassium (**C**) under different treatments from 2017 to 2019 during the experiment. Bars represent standard error. Different lowercase letters above the bars represent significant differences among treatments (*p* < 0.05).

Soil alkaline nitrogen (AN, Figure 2A), available phosphorus (AP, Figure 2B), and available potassium (AK, Figure 2C) contents decreased with soil depth. AN and AK contents varied among tillage practices in the 0–40 cm layer, while there was still a difference in AP in the 40–50 cm soil layer. Under treatment with the same tillage practices in maize season, the AN, AP, and AK contents increased by 6.11%, 0.22%, and 5.79%, respectively, under treatment with rotary tillage in 0–20 cm compared to deep tillage in the wheat season; while the AN, AP, and AK content decreased by 6.22%, 7.33%, and 6.59% in 20–40 cm, respectively. Under the same tillage mode in wheat season, the AN, AP, and AK contents were higher under treatments with subsoiling than under treatment with no-till. Compared with no-till in maize season, subsoiling combined with rotary tillage in wheat season increased the AN, AP, and AK contents in the topsoil (0–20 cm) by 12.47%, 5.47%, and 8.88%, respectively, while the relevant increment was 4.77%, 6.35%, and 2.40%, respectively, in the subsoil (20–40 cm) under the treatments with subsoiling combined with deep tillage in the wheat season.

#### 2.1.3. Soil Nitrate Nitrogen and Ammonium Nitrogen Contents

**Figure 3:**
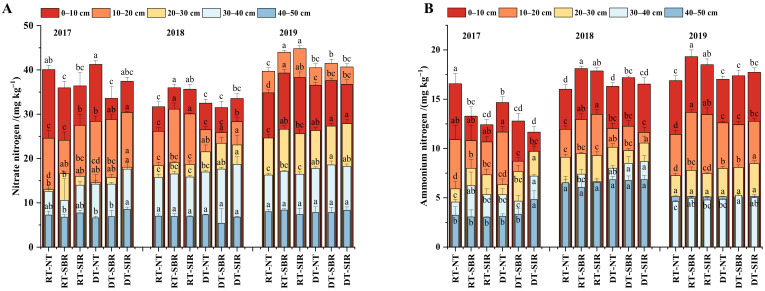
Soil nitrate nitrogen (**A**) and ammonium nitrogen (**B**) under different treatments from 2017 to 2019 during the experiment. Bars represent standard error. Different lowercase letters above the bars represent significant differences among treatments (*p* < 0.05).

Soil nitrate nitrogen (NO_3_^−^-N, Figure 3A) and ammonium nitrogen (NH_4_^+^-N, Figure 3B) contents were significantly influenced by tillage modes in the 0–40 cm layer, and both slightly increased with time. They were higher under treatments with no-till in maize season in the first year (2017), while they were lower in the following two years. In the topsoil (0–20 cm), the NO_3_^−^-N and NH_4_^+^-N contents were higher under treatments with rotary tillage than under treatments with deep tillage in the wheat season, while it was the opposite trend in the subsoil (20–40 cm). The NO_3_^−^-N and NH_4_^+^-N contents were higher under treatments with no-till in maize season than under treatments with subsoiling.

For the maize season tillage in 2017, compared with the treatments with subsoiling, the NO_3_^−^-N content at 0–10 cm and the NH_4_^+^-N content at 0–20 cm increased by an average of 13.65% and an average of 19.56%, respectively, under the treatments with no-till. However, the opposite trend was found in the 0–20 cm layer in 2018–2019, where the NO_3_^−^-N and NH_4_^+^-N contents under subsoiling were 7.35% and 7.39% higher, respectively than under no-till. At 20–40 cm, the NO_3_^−^-N and NH_4_^+^-N contents were higher under DT-SIR than under other treatments.

#### 2.1.4. The Interaction

The ANOVA results (Table 1) show that soil depth, year, treatment, and their interactions all had significant or highly significant differences in SOC, TN, C/N, AP, AK, NO_3_^−^N, and NH_4_^+^-N, but not in AN.

### 2.2. Principal Component Analysis of Soil Nutrient Indicators

**Figure 4:**
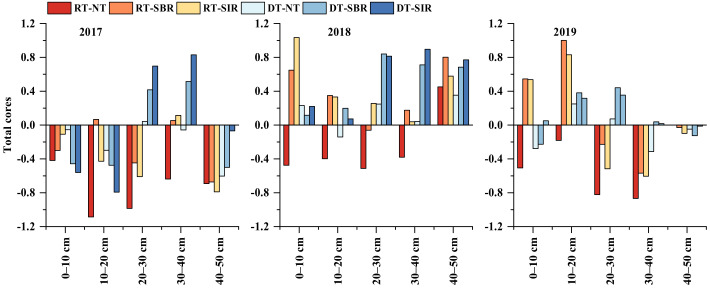
General score of soil nutrients during 2017–2019.

The PCA (Figure 4) showed 74.99–88.58% of the variability at 0–50 cm. The eigenvalues and the variance contribution rates for the different soil depths are reported in Table 2. The integrated score at 0–20 cm under RT-SBR and RT-SIR was higher than that under the other treatments, which indicated that the combination of rotary tillage in the wheat season and subsoiling in the maize season improved the soil nutrient content in the 0–20 cm soil layer. The integrated score under all treatments in the 0–10 cm soil layer was the highest in 2018. The positive and higher integrated scores in the 20–40 cm soil layer was under DT-SBR and DT-SIR and reached their peaks in 2018. Additionally, in the 40–50 cm layer, the integrated scores under all treatments were greatest and positive in 2018. Generally, the integrated scores under the treatments with rotary tillage in the wheat season and subsoiling in the maize season in the surface layer (0–20 cm), and the treatments with rotary tillage in the wheat season and subsoiling in the maize season in the subsurface layer (20–40 cm) showed positive values in 2018 and 2019.

### 2.3. Soil Ecosystem Multifunctionality

**Figure 5:**
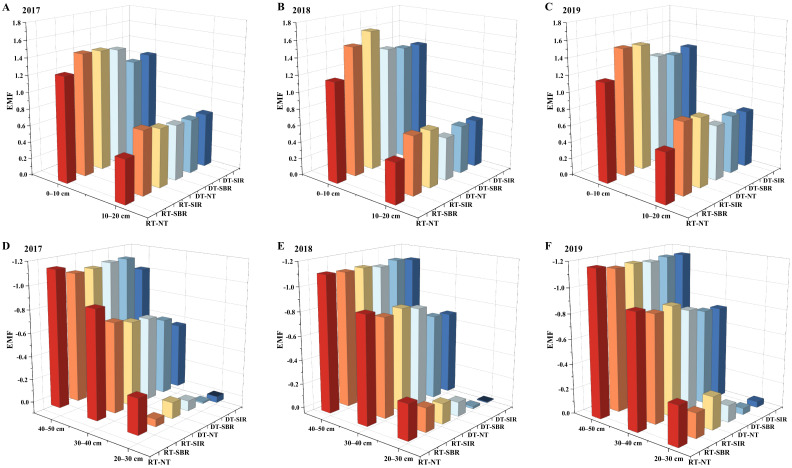
Soil ecosystem multifunctionality indexes in the 0–20 cm ((**A**): 2017, (**B**): 2018, and (**C**): 2019) and in the 20–50 cm ((**D**): 2017, (**E**): 2018, and (**F**): 2019) during 2017–2019.

The soil ecosystem multifunctionality (EMF) index decreased with increasing soil depth (Figure 5). Except for DT-NT in 2017, RT-SBR and RT-SIR treatments had higher EMF index values in the 0–20 cm soil layer during the three years. The EMF index in the 20–40 cm soil layer under the treatment with deep tillage in the wheat season was higher than that under treatment with rotary tillage; the highest EMF was under DT-SIR.

**Figure 6:**
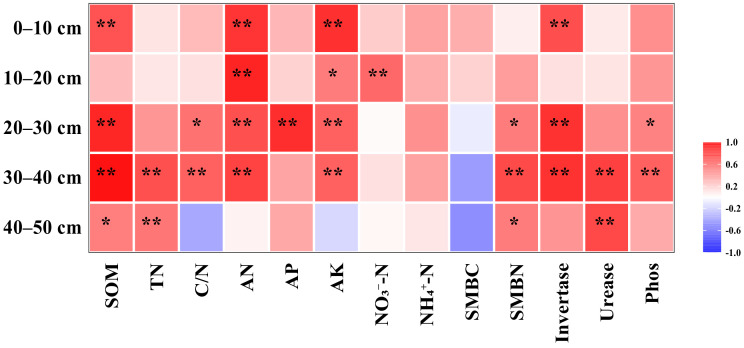
Plot of correlation coefficients between soil nutrient content and EMF indexes. * represents *p* < 0.05; ** represents *p* < 0.01.

Although the soil EMF index was positively correlated with soil nutrient content, there were obviously differences among the depths (Figure 6). More soil nutrient indexes at 20–40 cm correlate with the EMF index than at 0–20 cm or 40–50 cm. In the 0–20 cm soil layer, the soil EMF index was significantly correlated with SOM, invertase activity, and available nutrients. In the 20–40 cm soil layer, the EMF index showed significant or highly significant correlations with SOM, TN, C:N, AN, AP, AK, SMBC, and three enzyme activities. In the 40–50 cm soil layer, the EMF index showed significant or highly significant correlations with SOM, TN, SMBN, and urease activity.

### 2.4. Maize Yield, Yield Stability, and Coefficient of Variation

**Figure 7:**
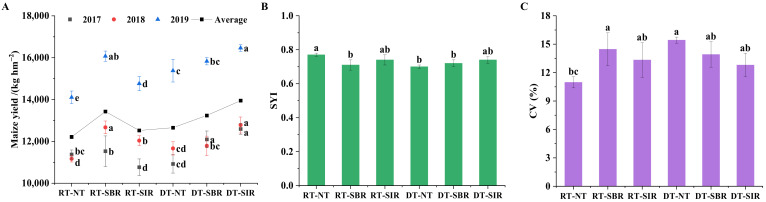
The maize yield (**A**), sustainability yield index (SYI, (**B**)), and coefficient of variation (CV, (**C**)) under different treatments. Different lowercase letters above the bars represent significant differences among treatments (*p* < 0.05).

The maize yield increased with time under each treatment. Generally, the subsoiling practice improved the maize yield compared with RT-NT (Figure 7). Moreover, maize yield was higher under DT-SIR during the three years. The smallest coefficient of variation (CV) and the largest sustainability index (SYI) were found under RT-NT, indicating that the yield under RT-NT fluctuated little and was more stable. Except for RT-NT, there were no significant differences in the CV and SYI of yields among treatments.

### 2.5. Relationship between the Soil Nutrient Content and Maize Yield

**Figure 8:**
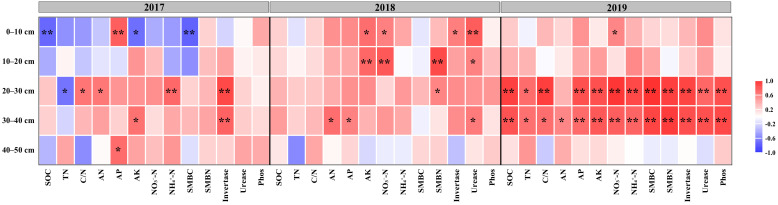
Plot of correlation coefficients between soil nutrient content and maize yield. * represents *p* < 0.05; ** represents *p* < 0.01.

The maize yield was affected by soil nutrients in different soil layers that changed with tillage time (Figure 8). In 2017, maize yield was positively correlated with main soil nutrients and invertase activity in the 20–30 cm soil layer. In 2018, maize yield was significantly or highly significantly positively correlated with tillage-layer AK, NH_4_^+^-N, SMBN, invertase, and urease activity. In 2019, there was an obvious correlation between soil nutrients at 20–40 cm and maize yield.

**Figure 9:**
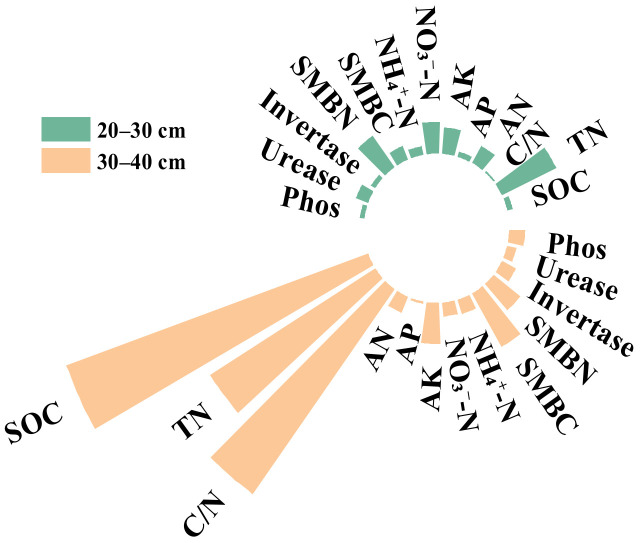
Contributions of soil nutrients to maize yield at 20–30 cm and 30–40 cm.

The contribution of soil nutrient factors to maize yield was higher at 30–40 cm than at 20–30 cm (Figure 9). Furthermore, SOC, C/N ratio, and TN were the key factors affecting maize yield in the 30–40 cm soil layer.

## 3. Discussion

The higher soil nutrient content at 0–20 cm was found under the treatments with rotary tillage in wheat season, while deep tillage was more effective in improving the nutrient content at 20–40 cm. The findings of this study showed that rotary tillage significantly impacts the topsoil, reducing soil weight and increasing soil porosity on the soil surface, which in turn helps to improve the soil microbial and enzymatic activity associated with soil nutrient cycling [34]. The plant residue, straw, and fertilizer are concentrated in the surface layer under rotary tillage, resulting in the enrichment of nutrients in the surface layer [35,36]. In contrast, deep tillage in the wheat season can break the hard bottom and deepen the cultivated horizon [17], which creates a better soil environment for microbes and increases soil enzyme activity, further promoting soil nutrient cycling. Deep tillage also helps to turn the straw and fertilizer from the surface layer into the deep soil layer, which provides the nutrient source for the deep soil layer and crop and further improves crop yield [6,16]. We further analyzed the tillage practices in maize season and found that, compared with no-till, the available nutrients (i.e., AN, NO_3_^−^-N, NH_4_^+^-N, AP, and AK) increased under treatments with subsoiling tillage at 0–50 cm. The main reason might be that subsoiling with straw return enhances soil macropores and total porosity and promotes the enzymatic activity and rhizosphere microorganism community [37,38]. Regular tillage is beneficial for distributing nutrients in the cultivated layer and enhancing soil productivity [39]. Studies have reported that minimal tillage and no-till are beneficial for the accumulation of organic matter and total nitrogen in the soil surface layer, while no-till combined with subsoiling can promote soil nutrient distribution in the surface and subsoil layer [40,41]. Deep tillage can significantly increase the active phosphorus content [17] and increase AN and AK in the 0–40 cm soil layer compared to no-till [42]. In this study, the AK content in the 0–20 cm layer under DT-SBR and DT-SIR had an overall increase with time during the three years. Such a trend can be attributed to the depth of residue placement in which incorporated residues increase SOM and AK contents due to accelerating decomposition caused by mechanical mixing of the soil [42]. Furthermore, deep tillage and subsoiling tillage contributed to a greater SOM content in the topsoil in the second year. The presence of mulch may have improved soil structure by stabilizing aggregates and protecting SOM against microbial degradation and reducing the rate of SOC decomposition [35,43,44]. According to the principal component score (Table 2), during the three-year experiment, the combined scores reached their peak in the second year, which indicated that the three-year rotation was conducive to the continuous improvement of soil nutrient content.

The soil EMF index is related to soil nutrient cycling, soil structural properties, and microbial activity [18,31] and is influenced by tillage practices. Our findings showed that nutrient content and enzyme activity in the topsoil layer were higher than those in the subsoil layer, but their correlation with the EMF index showed the opposite trend. This indicates that deep tillage and subsoiling can increase the ecosystem multifunctionality of the deeper soil layer. Studies have shown that soil nutrient effectiveness plays a crucial role in regulating the multifunctionality of soil EMF; both soil nutrient content and its stoichiometric ratio are important [45,46].

The higher crop yield was found under treatments with deep tillage in wheat season, which agreed with the reports from Han et al. [17] and Schneider et al. [47]. Deep tillage during the wheat season improves the physical properties of the soil and provides a better soil environment for maize root growth and development [48]. For maize season tillage, maize yields were higher with subsoiling tillage than no-till. Subsoiling tillage in maize season can improve water uptake during the filling stage, which facilitates maize grain filling [49], thus contributing to an increased crop yield. It has been found that suitable deep loosening tillage could break the bottom layer of the plow, improve the water storage capacity of the soil [50], facilitate the growth and development of maize roots [2,51], further increase maize yield, and contribute to the growth of the next crop [49,52,53]. Compared to conventional tillage, rotation tillage improved soil water storage [54] and promoted the accumulation of dry matter and maize yield [55]. Maize yield was highly correlated with soil nutrient content in the subsoil layer (20–40 cm) than that in the topsoil layer (0–20 cm) in the third year of this experiment. Maize root biomass is mainly concentrated in the 0–30 cm layer [56], and the subsoil layer is an important distribution layer for crop roots [57,58].

## 4. Materials and Methods

### 4.1. The Experimental Site

The field experiment was carried out in 2016 at Yuanyang, Henan Province, China (35°19′ N, 113°50′ E). The region has a sun-humid continental monsoon climate, with an annual mean temperature of 14.5 °C and annual precipitation of 615 mm. The soil type is fluvo-aquic soil, which is Calcaric Cambisol according to WRB [59]. The initial soil properties were soil organic matter (SOM) of 17.3 g kg^−1^, total nitrogen (TN) of 1.0 g kg^−1^, alkaline nitrogen (AN) of 71.3 mg kg^−1^, available phosphorus (AP) of 21.6 mg kg^−1^, available potassium (AK) of 108 mg kg^−1^, and a pH of 7.2.

### 4.2. Experimental Design

**Figure 10:**
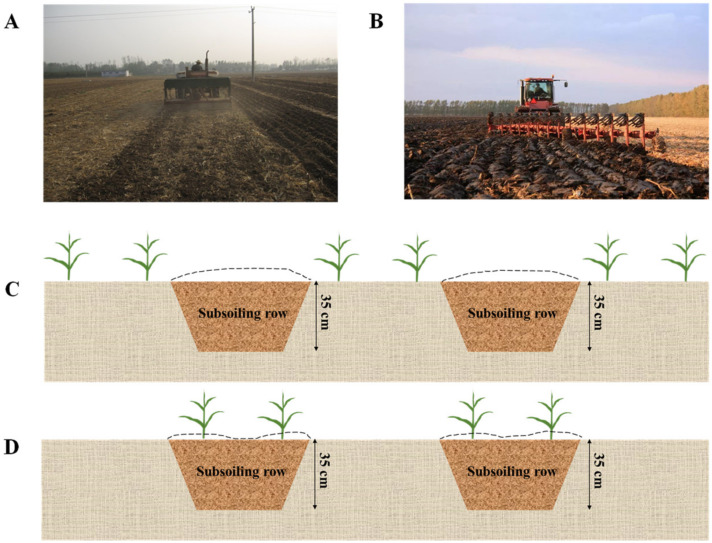
Scheme representative of the tillage management: (**A**) rotary tillage in wheat season, (**B**) deep tillage in wheat season, (**C**) inter-row deep tillage in maize season, and (**D**) intra-row deep tillage in maize season.

The split-plot design with rotary tillage and deep tillage in the wheat season as the main treatment and no-tillage, inter-row deep tillage, and intra-row deep tillage in the maize season as the vice treatment was set with a total of six treatments: (1) Rotary tillage–no tillage (RT-NT); (2) Rotary tillage–subsoiling inter-row (RT-SBR); (3) Rotary tillage–subsoiling in row (RT-SIR); (4) Deep tillage–no tillage (DT-NT); (5) Deep tillage–subsoiling inter-row (DT-SBR); (6) Deep tillage–subsoiling in row (DT-SIR). Each treatment was repeated three times. The wheat and maize straws were returned after harvest. In the wheat season, the rotary tillage depth was 13–15 cm, and the deep tillage depth was 28–30 cm; in the maize season, the subsoiling depth was 35 cm (Figure 10). The row widths of subsoiling were 61.3 cm, and the maize plant’s space was 21.9 cm. The cropping system was a winter wheat–summer maize system. Winter wheat and summer maize were planted at densities of 232.5 kg hm^−2^ and 67,500 plants hm^−2^, respectively. The base fertilizers were 150 kg N hm^−2^, 120 kg P_2_O_5_ hm^−2^, and 120 kg K_2_O hm^−2^ before winter wheat sowing, followed by 69 kg N hm^−2^ at the wheat jointing stage; single basal fertilizers were 210 kg N hm^−2^, 75 kg P_2_O_5_ hm^−2^, and 90 kg K_2_O hm^−2^ before summer maize planting.

### 4.3. Sampling and Analysis

The soil sample was randomly collected after the maize harvest in 2017–2019 from each plot as a composite sample with 5–10 cores (5 cm in diameter). The 0–50 cm depth soil was collected at 10 cm intervals. The soil samples were divided into two parts; the fresh part was stored at 4 °C to determine soil microbial biomass carbon (SMBC) and nitrogen (SMBN), nitrate nitrogen (NO_3_^−^-N), and ammonium nitrogen (NH_4_^+^-N); the other part was air-dried and sieved to determine soil nutrient content. SOM was measured using the potassium dichromate oxidation method; TN was measured using the Kjeldahl digestion method; AN was determined by the NaOH alkali diffusion method; AP was determined by the Olsen method; AK was determined by the fame photometric method; NO_3_^−^-N and NH_4_^+^-N were obtained using the colorimetric method, according to Bao [60]. SMBC and SMBN were measured using the chloroform fumigation–extraction method [61,62]. The invertase, urease, and neutral phosphatase activity were determined by the 3,5-dinitrosalicylic acid colorimetric method, indophenol blue method, and the disodium phenyl phosphate colorimetry method, respectively, according to Guan [63].

At the maize maturity stage, three rows of maize (15 m in length in every harvested row) were harvested from each plot. The total number of ears harvested in each plot was recorded. The number of grains and rows per ear and the length per ear of harvested maize were measured before threshing. The fresh weight of the grain was determined before drying to a constant weight in an oven at 75 °C, and after oven drying weighed it again. The dry 100-grain weight was measured. The maize yield was calculated according to the national maize grain warehousing standard (at a moisture content of about 14%).

### 4.4. Calculations

We used thirteen soil indexes (SOM, TN, C:N, AN, AP, AK, NO_3_^−^-N, NH_4_^+^-N, SMBC, SMBN, invertase activity, urease activity, and neutral phosphatase activity) to assess soil ecosystem multifunctionality (EMF). They were standardized by Z-Score [64], and the average value was calculated as the EMF index. The calculation formula is below:

(1) $$Z-\mathrm{score}=(x-{\mathrm{mean}}_{i})/{\mathrm{SD}}_{i}$$

in which x is the soil index, mean_i_ is the average of soil index i, and SDi is the standard deviation of soil index i.

The sustainable yield index (SYI) [65,66] and coefficient of variation (CV) [67] were used to evaluate yield sustainability and yield stability. The higher the SYI value, the better the yield sustainability; the lower the CV value, the better the yield stability:

(2) $$\mathrm{SYI}=(\bar{Y}-\sigma )/Y_{\max}$$

(3) $$\mathrm{CV}=\sigma /\bar{Y}✕100\%$$

in which $\bar{Y}$ is the average yield (kg hm^−2^), σ is the standard deviation (kg hm^−2^), and Y_max_ is the highest yield (kg hm^−2^).

### 4.5. Statistical Analysis

The data was collated and analyzed using Excel 2016 and SPSS 20.0 statistical software (SPSS, Inc., Chicago, IL, USA). The treatment differences were analyzed using the least significant difference test (LSD) at *p* ˂ 0.05. Correlations were calculated using the Pearson coefficient. Principal component analysis (PCA) was used to extract three principal components according to the principle of eigenvalue > 1 as the principal component extraction, and scores were calculated to evaluate soil fertility quality. The graphs were created using Origin 2021 (Origin Lab Corporation, Northampton, MA, USA).

## 5. Conclusions

The findings from the three-year experiment showed that the treatments with rotary tillage in wheat season increased the soil nutrient content and EMF index in the 0–20 soil layer. Conversely, for SOM, TN, AN, AP, NO_3_^−^-N, and NH_4_^+^-N contents in the 20–40 cm soil layers under treatments with deep tillage in the wheat season. Meanwhile, regardless of rotary tillage or deep tillage in wheat season, the soil nutrients and EMF index at 0–40 cm were higher under the treatment with subsoiling than with no-till in maize season. Moreover, maize yield under treatments with deep tillage in the wheat season increased by an average of 4.53% compared with that under treatments with rotary tillage; on the other hand, the maize yield under treatments with subsoiling in the maize season was an average of 6.88% higher than that under treatments with no-till in the maize season. The correlation analysis demonstrated that maize yield was closely related to the soil nutrients at 20–40 cm. Moreover, the SOC, TN, and C/N played important roles in maize yield. Generally, the combination of deep tillage in wheat season and subsoiling in maize season was conducive to improving the soil nutrient content by 5.39% on average in the subsoil; meanwhile, the maize yield was enhanced by 5.71% on average. Considering the cost, it is recommended to utilize this tillage practice every three years in this area.

## Figures and Tables

**Table 1:** The interaction of soil depth, experimental years, and treatment with soil properties.

Source of Variation	SOM	TN	C/N	AN	AP	AK	NO_3_^−^-N	NH_4_^+^-N
Depth	7087.83 ***	7363.19 ***	1012.51 ***	3035.45 ***	5305.48 ***	5733.86 **	5182.04 ***	3697.76 ***
Year	88.69 ***	925.08 ***	86.10 ***	6.26 ***	25.78 ***	47.99 ***	622.36 ***	502.33 ***
Treatment	32.57 ***	14.06 ***	15.19 ***	12.51 ***	16.29 ***	18.10 ***	16.74 ***	9.52 ***
Depth × Year	13.33 ***	119.56 ***	29.52 ***	14.34 ***	4.65 ***	72.94 ***	171.68 ***	61.88 ***
Depth × Treatment	12.35 ***	10.19 ***	5.80 ***	5.07 ***	2.22 **	13.95 ***	7.03 ***	9.81 ***
Year × Treatment	5.03 ***	6.34 ***	3.85 ***	1.24 NS	4.14 ***	2.36 *	7.20 ***	5.90 ***
Depth × Year × Treatment	2.48 ***	5.18 ***	2.24 ***	0.90 NS	3.51 ***	3.07 ***	4.90 ***	7.13 ***

The source of variation: “Depth” means the five sample depths; “Year” means the tillage duration; “Treatment” means the six tillage modes. * represents *p* < 0.05; ** represents *p* < 0.01; *** represents *p* < 0.001; NS represents *p* > 0.05.

**Table 2:** Eigenvalues and variance contribution rates.

Soil Depth	Factor	PC1	PC2	PC3
0–10 cm	Eigenvalues	2.778	2.185	1.036
	Percent (%)	34.729	27.314	12.946
	Cumulative (%)	34.729	62.043	74.989
10–20 cm	Eigenvalues	3.484	2.11	1.219
	Percent (%)	43.544	26.38	15.239
	Cumulative (%)	43.544	69.924	85.163
20–30 cm	Eigenvalues	3.597	2.403	1.087
	Percent (%)	44.956	30.034	13.59
	Cumulative (%)	44.956	74.991	88.58
30–40 cm	Eigenvalues	3.355	1.967	1.211
	Percent (%)	41.939	24.59	15.14
	Cumulative (%)	41.939	66.528	81.668
40–50 cm	Eigenvalues	3.283	2.28	1.111
	Percent (%)	41.035	28.505	13.889
	Cumulative (%)	41.035	69.539	83.429

## Data Availability

Not applicable.
